# Correction: Role of microglia in the dissemination of Zika virus from mother to fetal brain

**DOI:** 10.1371/journal.pntd.0009344

**Published:** 2021-04-13

**Authors:** Pei Xu, Chao Shan, Tiffany J. Dunn, Xuping Xie, Hongjie Xia, Junling Gao, Javier Allende Labastida, Zou Jing, Paula P. Villarreal, Caitlin R. Schlagal, Yongjia Yu, Gracie Vargas, Shannan L. Rossi, Nikos Vasilakis, Pei-Yong Shi, Scott C. Weaver, Ping Wu

The fourteenth author’s name is spelled incorrectly. The correct name is: Nikos Vasilakis.

## References

[1] Xu P, Shan C, Dunn TJ, Xie X, Xia H, Gao J, et al. (2020) Role of microglia in the dissemination of Zika virus from mother to fetal brain. PLoS Negl Trop Dis 14(7): e0008413. 10.1371/journal.pntd.0008413 32628667 PMC7365479

