# Supplementary material for: Role of microglia in the dissemination of Zika virus from mother to fetal brain
Source: PLoS Negl Trop Dis. 2020 Jul 6;14(7):e0008413. doi: 10.1371/journal.pntd.0008413 (PMC7365479; doi:10.1371/journal.pntd.0008413)
Supplement: Attachment — Submitted filename: Response-to-Reviewers_final.doc [file pntd.0008413.s001.doc]

**Reviewer #1:**

1. We have no concerns about the methods used in this study. We would like to encourage the authors to measure infectious virus and not just viral RNA as this does not confirm whether use of the monoclonal antibody actually prevents virus replication in tissues.

According to the reviewer’s suggestion, we performed a plaque assay to measure the infectious ZIKV released from the infected yolk sac (YS) microglia. The data is now added in S3 Fig (see also our response to Reviewer #2, comment #4 below). On the other hand, we were able to determine ZIKV RNA copies in the infected tissues by RT-qPCR based on a standard curve produced from serial dilutions of the infectious viral stock with a known concentration (**page 6, lines 106-108**). Further clarification on the effect of the anti-CSF1R monoclonal antibody is included in Discussion (**page 17, lines 360-365**)

2. The fundamental missing piece of data that results in an incomplete story is whether treatment of dams with anti-CSFIR reverses the detrimental effects of ZIKV on offspring viability (as shown in Figure 1). It is never reported whether by reducing ZIKV in the brain microglia reverses the adverse effects of ZIKV on development or if that is caused by something else (e.g., immunopathology). Along those , lines, the authors also do not show the impact of anti-CSFIR alone (without ZIKV) on microglia and adverse outcomes. Finally, there are no data pertaining to microglia activation, only co staining for ZIKV. Does anti-CSFIR reduce not only microglia infection but activation (i.e., neuroinflammation)?

We agree with the reviewer and now include the additional data in S4 Fig A. Basically, we found that anti-CSF1R reduced ZIKV-caused fetal demise. This is also clarified in Results (**page 13, lines 255-256**).

Toward our main goal for the role of microglia in ZIKV vertical transmission, the anti-CSF1R was used to temporarily deplete yolk sac microglia but not to reduce ZIKV infection in microglia. Since anti-CSF1R efficiently ablate microglia (over 99%) in both yolk sac (YS) and brain, the inflammatory status of microglia per se may have minimal if any effect on brain development. As pointed by the reviewer, however, it would be interesting to determine whether anti-CSF1R could directly reduce ZIKV infection/replication in microglia. Although depleting microglia reduces ZIKV RNAs in embryo brains, we do not expect that this is an antiviral strategy to reverse the adverse effects of ZIKV on brain development simply because microglia themselves play an important role in the brain development. Further studies would be needed to explore how mitigating microglia-associated ZIKV transport leads toward the development of new interventions. Further clarification is now added in Discussion (**page 17, lines 345-365**).

**Reviewer #2:**

1. The data in figure 1B are very interesting, though the experiment is under-powered with only an n of 4. This experiment should be repeated. To what do the authors attribute the differences in maternal weight gain following early term infection vs late term infection? Importantly, does the virologic status of the dam change depending on the gestational timepoint of infection? These data would clarify the authors’ overall findings considerably and bolster this study’s value as a reference for the field.

We have added more data in the revised Figure 1B with the number of dams in each group increased to 6.

The dams infected during early embryonic stage (E6.5-8.5) did not gain as much weight as those infected later (E9.5 to E15.5). This is most likely because more embryos following early infection were resorbed or abnormally developed. This is further clarified in Results (**page 8, lines 147-150**)

There were no significant differences in the viral load of dam cortex and dam serum between early and late infection (new data is now added to S1 Fig B, and further clarified in Results (**page 8, lines 156-157**).

2. While the images in Figure 2 are generally of high quality, the reliance on representative images with no quantification diminishes enthusiasm for the study. Quantification of the numbers of infected F4/80+ cells detected per unit area in yolk sac and fetal brain should be provided to establish the level of variability and overall generalizable nature of these findings.

Following the reviewer’s comment, we now add the quantitative data in Fig 2 (D and G). With these and other additional quantitative data, the original Fig 2 is now split into two figures (Fig 2 and Fig 3)

3. In Figure 2C, how were the imaged structures determined to be blood vessels? Why do F4/80+ cells appear to permeate the yolk sack in 2B, but seem concentrated in a single blood vessel in 2C. The differences in cellularity between these two images is confusing. Again, more robust quantification and explanation of analytical methods is necessary to interpret these data.

Thanks for pointing out the YS issue. We now include additional data as Fig 2B and further clarify the issue in Results (**page 10, lines 192-194**). Briefly, we found that normal YSs were full of vessel-like structures with a pink color; whereas ZIKV-infected YSs exhibited a reduced number of vessels with yellowish or brownish color, indicating some tissue damage (Fig 2B).

As suggested, data are now quantified and analyzed. In control YS, the F4/80^+^ cells are widely distributed. However, the number of F4/80^+^ cells is reduced in the infected YS, especially in ZIKV-stained areas, indicating ZIKV-mediated damage or reduction of cell genesis. We also removed the description of “vessel” in Fig 2C, and added S2 Fig A to show the ZIKV^+^ F4/80^+^ cells in the YS and connecting vessels (determined anatomically). These are further clarified in Results (**page 10, lines 194-196**).

4. A more quantitative assessment of viral infection in YS microglial progenitors is warranted in Figure 2E. Are these cells productively infected, producing progeny virus? What is the time course of viral burden and shedding? Multistep growth curve or similar analyses would be helpful to more robustly profile the virologic profile of these cells.

Data were quantified and analyzed. The n value for embryos and number of unique litters are reported. Plaque assays were done to determine the viral burden and shedding. Data is now added as S3 Fig, and further clarified in Results (**page 11, lines 227-228**).

5. The efficiency of depletion of microglia in YS and fetal brain following antibody administration to the pregnant dam is somewhat surprising. The authors should include quantification of F4/80+ cells per unit area across multiple mice in figures 3B and 3C to establish the consistency and reproducibility of this depletion.

Data were quantified and analyzed (now becoming Fig 4C and E). Our data are consistent with the 99% ablation efficiency previously reported by Squarzoni et al (2014) and Hoeffel et al. (2015). The n value for embryos and number of unique litters are now added. Further clarification is also made in the text (**page 12, lines 247-251**)

6. Were microglia also depleted in the brains of dams treated with anti-CSFR1 antibodies? While in theory, perhaps, these antibodies would not cross the BBB, this should be experimentally confirmed. If changes to maternal microglial numbers are discovered, these should be accounted for in the interpretation of the authors’ results.

Easley-Neal et al (2019) reported that these antibodies can cross the blood-brain barrier through intraperitoneal injection in adult mice to deplete microglia in the adult brain. Our data show lack of ZIKV-induced changes in the dam brain 3-5 days after anti-CSF1R antibodies treatment, indicating that the antibody is unlikely affecting viral replication in the dam brain in our system (Fig 4I). Further clarification is now included in the text (**page 17, lines 360-365**).

7. The rationale for reporting miscarriage rates in Ifnar1xWT vs. Ifnar1xIfnar 1 crosses (Figure S1) is unclear. Are the authors justifying why they chose to use WT sires for their main studies? At present, these data do not seem relevant and need further discussion. Moreover, these data are contradictory to those reported by Yockey et al (PMID 29305462) who reported worse outcomes in Ifnar1-heterozygous embryos compared to Ifnar1-deficient embryos following ZIKV infection. The authors should discuss this discrepancy.

It is indeed the initial finding of different miscarriage rates in crossing IFNAR1 null dams with WT vs. IFNAR1 null sires that informed our design to use the former pairing strategy for the main studies. The choice of using WT sires allowed us to generate enough embryos effectively to address the critical issue of the role of microglia during ZIKV infection and embryonic brain development. In addition, the miscarriage rate of our Ifnar1−/− x WT model is closer to that of the nonhuman primates model (Dudley et al, 2018). Further clarification is now added to Results (**pages 7-8, lines 139-145**) and Discussion (**page 14, lines 285-289**).

We thank the reviewer for pointing out the literature by Yockey et al (2018) who found that Ifnar1−/− fetuses had a higher survival rate than Ifnar1+/− littermates. First, Yockey et al. did not report the miscarriage rate in their study. Second, we have similar findings in terms of fetal survival. These are further clarified in Discussion (**page 14, lines 289-293; page 18 lines 384-385**).

8. Similar to above – what is the purpose of Figure S2? Are the authors justifying using an infectious clone in their study? These data should be more well integrated into the paper or removed.

Accordingly, the original Fig S2 has been removed.

9. The statistical comparisons in Figures 1G-I appear inconsistent. Some comparisons with smaller effect sizes and higher variance have higher p values than other comparisons with larger effect sizes and less variance. Which test was used for these graphs, specifically? For Figure 1I, why are values reported below the limit of detection?

As suggested, all data from Fig 1G-I are now analyzed by the non-parametric Kruskal Wallis test with Dunn’s multiple comparisons, this information is clarified in the Fig 1 legend.

When using RT-qRCR to measure viral RNAs, nonspecific background signals could be detected in some tissue samples. Intact control samples should have zero RNA copies. However, sometimes there is still a readout from the RT-qPCR machine with high CT value (>35), which yields a value when converted to RNA copies. Consequently, a so-called “limit of detection” is arbitrarily set, and all mock-infected samples are indeed below or equal to this arbitrary detection level.

10. Funk and colleagues recently reported decreased virologic control of WNV-infected mice treated with a CSFR1 antagonist (PMID 30704498). In general, more detailed discussion of previous studies demonstrating the anti-viral capacity of microglia in adults using similar depletion strategies should be discussed, as they contrast somewhat with the authors findings in fetuses in the current study.

Agree with the reviewer, clarification of this discrepancy is now included in Discussion (**pages 17-18, lines 366-377**).

11. A major issue of concern is the use of immunodeficient Ifnar-/- animals as the basis for in vivo phenotypes. IFN-deficient mice exhibit greatly increased viremia and expanded viral tropism that may confound the ability to truly map routes of entry into the fetal CNS as they occur naturally. This issue is one faced by the entire field and not solely a problem with the current manuscript, but some circumspection in the interpretations of the authors’ findings is warranted in the discussion. While likely beyond the scope of the current manuscript, establishing similar phenotypes in an immunocompetent mouse model (such as the recently developed hSTAT2 knockin mouse) or even in NHP models will be needed to confirm the authors’ findings. The authors should elaborate on these points in the discussion.

According to the reviewer’s suggestion, further discussion emphasizing the limitations of this model is now added (**pages 18-19, lines 378-395**).

12. While an intriguing hypothesis, the authors do not currently have sufficient evidence to establish any role for microglial progenitors in “vertical transmission.” Any virus present in the fetal YS would in theory already be “transmitted” from the infected dam. Instead, the authors posit a possible route of viral dissemination to the fetal brain, but even this possibility will need more careful experimentation to establish. The authors findings could simply arise from a depletion of a tropic cell type from the fetal brain, thereby diminishing a major replicative niche for the virus, irrespective of the movement of infected cells from YS to fetal brain. The authors should be more circumspect in their interpretation of the findings in figure 3, or otherwise perform more careful time course experiments in which YS microglia are depleted either before or after populating the fetal brain.

As the reviewer suggested, we changed the title from vertical transmission to dissemination. In addition, we clarified the interpretation of the findings in discussion (**pages 15-16, lines 306-344**).

13. For the n values for embryos throughout the manuscript, the number of unique litters should also be reported.

Changes are made throughout the manuscript according to the suggestion. The n values were reported in the figure legends (e.g. **page 9, lines 174-180**).

14. In this study, Xu et al present intriguing evidence for the importance of gestational age on the dissemination of ZIKV to the fetal brain, with some exploration of possible roles for yolk sac derived microglia. The authors’ systematic characterization of the virologic outcomes of maternal ZIKV infection at different stages of gestation is highly informative and an important addition to the existing literature. While the authors convincingly demonstrate that fetal microglial progenitors are tropic for ZIKV and contribute to viral burden in the fetal brain, the purported roles for these cells in transmission/dissemination are somewhat prematurely drawn. While this study will likely be of broad interest, some improvements are warranted before publication.

More careful interpretation as the reviewer suggested is now added to Discussion (**pages 15-18, lines 306-377**).

**Reviewer #3:**

1. Please include more information on the sample size for each experiment.

The number of embryos per group (n) and the number of litters are now included for each experiment (in results and figure legends).

2. Additional information on how representative the images shown are and if there is any way to quantify the level of ablation among the different animals it would strengthen they results.

As suggested, the quantitative analyses of images related to the level of ablation are added in Fig 4C and E.

3. Fig 2B – Add a scale bare to the zoomed images.

The scale bar is now added to Fig 2C.

4. Fig 2 – Please add the number of embryo brains processed that showed similar data.

The n value for embryos and the number of litters per group are now added to figure legend and results. Similar data are shown in S2 Fig B.

5. Fig 3 – How consistent was the ablation of YS microglia? Only a single micrograph is shown, is this representative of X embryos, or did some embryos retain higher levels of staining? Is there any way to quantify (RT-PCR in embryo brain?)?

Quantitative analyses are now added in the new Fig 4C and E. Briefly, we found that application of anti-CSF1R during the early stage of YS microglial-genesis (E6.5-E7.5) consistently and efficiently deplete microglia in both YS (over 99%) and embryo brain (over 90%). Our data is consistent with the 99% ablation efficiency previously reported by Squarzoni et al. (2014) and by Hoeffel et al. (2015). Further clarification is now added to Result (**page 12, lines 247-253**).

6. I assume ablation of microglial progenitor cells during this early time of development has severe consequences to normal embryo brain development. Please discuss how this alteration in brain development could alter ZIKV replication in the brain. What would happen if you ablated the cells and directly inoculated the brain? Would there be less permissive cells there? To further establish these cells are a “Trojan horse” for the virus to get across the maternal-fetal interface, can you quantify virus levels in non-brain tissue of the embryos? Is it similarly decreased or is it specific for virus levels in the brain?

We agree with the reviewer, and have added to the Discussion (**page 17, lines 345-358**). Briefly, microglia can modulate normal embryonic brain development by affecting the proliferation of neural stem/progenitor cells (NS/PCs), particularly during the peak of NS/PC proliferation and differentiation. Thus, ablation of microglia could, in theory, reduce the number of NS/PCs and consequently the ZIKV RNA load. However, microglia started to migrate into embryo brains at E9-10, and the effect of microglia ablation on NS/PC proliferation by the time we collect the embryos (E11.5) is likely minimal. The reduced numbers of microglia and, maybe, NS/PCs will not count for the degree of ZIKV RNA reduction in the embryo brains.

We have checked the viral levels in non-brain tissues of embryos, and found that anti-CSF1R treatment at E6.5-7.5 also reduced ZIKV RNA copies in the heart (data is now added as S4 Fig B, and further clarified in **page 13, lines 256-258**). Since CSF1R^+^ erythro-myeloid progenitor-derived primitive macrophages seed into all fetal tissues and YS developed before a fully functional placenta, our data may indicate a role of YS erythron-myeloid progenitors and their differentiated microglia and macrophages as “Trojan horse” in the vertical transmission of ZIKV. Further studies in additional rodent and non-human primate models are warrant.

7. Fig 1B – Figure is labeled “WT”, but I think it is showing “mock” infected data?

It is indeed the mock, and typo is now corrected.

8. Fig 2A – The Figure legend description of the pictures is somewhat hard to follow. There are several arrows in the picture and two things circled. It may help to number each embryo. Alternatively, if the picture is shown to demonstrate the distinct location of the yolk sac versus the embryo you may want to just include a cartoon and last picture in the series.

Fig 2A with the relevant figure legend are revised. Photos of embryo with the outside yolk sac and placenta are shown in Fig 2B and E.

9. This study by Xu et al is characterizing the role of yolk sac microglial progenitor cells in transporting Zika virus across the maternal placenta to infect the developing embryo. Ablation of the yolk sac microglial progenitor cells reduced ZIKV in the embryonic brain, although did not eliminate it. The paper is well written and straight forward. To enhance the conclusions of the study additional discussion about how ablation of the YS microglial progenitor cells will alter the brain structure is warranted.

According to the suggestion, additional clarification is now added in Discussion (**pages 17, lines 345-358**). See also our response to this reviewer in #6 above.
