# Supplementary material for: Role of microglia in the dissemination of Zika virus from mother to fetal brain
Source: PLoS Negl Trop Dis. 2020 Jul 6;14(7):e0008413. doi: 10.1371/journal.pntd.0008413 (PMC7365479; doi:10.1371/journal.pntd.0008413)
Supplement: Attachment — Submitted filename: Response_to_Reviewer.docx [file pntd.0008413.s002.docx]

**Reviewer #1:**

1. The authors have cultured Yolk sac microglia-progenitors, infected them with ZIKV, and then performed a plaque assay. We had intended that the authors would have use a plaque assay or similar measure of infectious virus to evaluate the infectious virus in collected tissue, for example in Figure 1G-I, and Figure 4F-J. In figure 4 specifically, this would allow the authors to show that the Anti-CSFR1 antibody decreased the amount of infectious zika virus in the embryonic brain. The plaque assay experiment in Figure S3 may fit better incorporated into figure 3.

In agreement with the reviewer, it would be ideal to perform the plaque assay on all tissues for infectious virus. However, based on our previous reports (Fontes-Garfias CR et al., 2017 Cell Rep), the mass amount of each tissue (mouse brain ~0.01 g at E11.5 and ~0.06 g at E18.5, and yolk sac 0.005 g at E11.5) during the embryonic stage for each mouse embryo and the viral load per gram of tissue are far below the limit of detection (or the sensitivity) of the plaque assay.

As suggested, Figure S3 is now moved to Fig 6D.

2. The authors’ additional experiment (Figure S4A) have addressed our main concern here, and have shown that depletion of the yolk sac progenitors with the anti-CSF1R antibody reduces the fetal demise during zika infection. This figure seems vital to the overall narrative of the manuscript, and we would suggest adding it to figure 4.

According to the reviewer’s suggestion, Figure S4A is now moved to the new Fig 7B.

3. Move data from supplemental information to main figures in text. Also, measuring infectious virus in tissues is needed.

All supplemental information is now moved to the main text. In terms of measuring infectious virus in tissues, please see our response to #1 above.
